# Organisational support and turnover intentions: A moderated mediation approach

**DOI:** 10.1002/nop2.911

**Published:** 2021-05-12

**Authors:** Saeed Pahlevan Sharif, Ester Ellen Trees Bolt, Ashraf Sadat Ahadzadeh, Jason James Turner, Hamid Sharif Nia

**Affiliations:** ^1^ Taylor’s Business School Taylor’s University Subang Jaya Malaysia; ^2^ Department of Journalism University of Xiamen Sepang Malaysia; ^3^ University of Technology & Innovation Kuala Lumpur Malaysia; ^4^ School of Nursing and Midwifery Amol Mazandaran University of Medical Sciences Sari Iran

**Keywords:** Iran, job satisfaction, nurses, organisational support, psychological ownership, turnover intention

## Abstract

**Aim:**

The current study aims to examine the moderating role of psychological ownership in the process that translates organisational support into nurses’ turnover intentions through job satisfaction.

**Design:**

A cross‐sectional research design was used to test the hypotheses.

**Method:**

Using a purposive sampling 341 self‐completed survey data were collected from nurses working in two public hospitals in Iran. Structural equation modelling was used to analyse the data.

**Result:**

The research revealed that organisational support and job satisfaction were negatively related to a healthcare professionals’ turnover intention. Moreover, job satisfaction mediated the negative relationship between organisational support and turnover intention. The research also revealed that psychological ownership strengthened the positive relationship between organisational support and job satisfaction.

## 1 INTRODUCTION

Labour turnover has been a widely discussed concept in the field of psychology, economics and organisational behaviour in recent years. It is however no surprise that academics and practitioners have been so interested in understanding why employees decide to voluntarily leave their organisations due to its multi‐faceted impact. Research has shown that labour turnover negatively impacts customer service (Hancock et al., 2013) and organisational performance (Park & Shaw, 2013). Moreover, there is growing evidence that labour turnover increases organisational costs through the need for recruitment and training (Allen et al., 2010), disturbing team dynamics (van der Vegt et al., 2010), and potential loss of organisational knowledge (Duffield et al., 2014).

Investigating factors contributing to labour turnover and turnover intention over the last century has resulted in the development of several theories to explain this phenomenon (Hom et al., 2017; Rubenstein et al., 2015). However, the underlying mechanism behind these relationships is still relatively limited with the concept largely unexplained (Lee et al., 2008). Research has shown continuous support for the positive direct relationship between turnover intentions and actual turnover (Griffeth et al., 2000; Rubenstein et al., 2018). Also, previous research reveals that employees with turnover intentions may already demonstrate deviant behaviours such as increased absenteeism, lateness and less interest in participating in training, which could affect their performance and in turn the organisation's performance (Mai et al., 2016). These issues highlight the importance to study the antecedents of turnover intentions.

From a theoretical perspective, organisational support and working conditions have been observed to be an influencer in employee's outcomes dating back to the 1920s when the Hawthorne experiment was conducted (Mayo, 2004). Labour turnover theories (Lee & Mitchell, 1994; Price, 1977; Steers & Mowday, 1981), empirical studies (Griffeth et al., 2000; Li et al., 2020; Lundmark et al., 2021; Pahlevan Sharif et al., 2018) and the results of meta‐analysis studies (Rhoades & Eisenberger, 2002) have highlighted the importance of employees’ working environment in explaining the employees’ turnover process. However, despite the plethora of studies supporting the direct relationship between organisational support and turnover intentions, an unresolved issue in these studies remains, which is the process that explains this association.

This study makes three main contributions to the existing literature. First, the current study addresses a call for testing of more complex models by investigating underlying mechanisms that drive labour turnover (Rubenstein et al., 2018). More specifically, the current study examines a moderated mediation model by proposing psychological ownership as the moderator and job satisfaction as the mediator in the relationship between organizational support and turnover intention. Psychological ownership refers to the “employee's personal feeling of possessiveness and of being attached to a person or object that makes the employee consider it as mine” (Pierce et al., 2001, p. 299). The identification of employees with an organization is in line with social identity theory (Tajfel & Turner, 1986). Individuals with high psychological ownership identify themselves with their job and organisation more, so it is expected that this strengthens the negative relationship between organisational support and job satisfaction. Despite theoretical support, empirical research on the role of psychological ownership in the relationship between organisational support, job satisfaction and turnover intention is scarce.

Second, this study investigates labour turnover of nurses in healthcare organisations which has become a global concern since high nurses’ turnover has been found to impact the quality of care provided to patients (Van Bogaert et al., 2010), results in longer patient hospital stays, increased job demands resulting in burnouts as well as an increase in recruitment costs (Duffield et al., 2014; Jones, 2008). These consequences could impact the healthcare organisations’ performance in meeting the demands of patients (Shields & Ward, 2001).

Third, in response to the call for further research to examine the cross‐cultural nature of labour turnover (Rubenstein et al., 2018), this study has been conducted in Iran, a country in which healthcare organizations face resource scarcity due to global sanctions. After the Islamic revolution in Iran in 1979, the government ruling has allowed and incorporated the principles of the Islamic religion on the state (Wright, 2010). Therefore, the Islamic revolution influenced the way businesses were operating, since all operations now had to be conducted according to the Islamic principles (Pahlevan Sharif et al., 2018). In response to the Islamic revolution in Iran, the country was subjected to sanctions by Western countries, such as the United States and later the United Nations, which impacted the Iranian people as well as the business environment, also affecting healthcare organizations due to resource scarcity. Although not all sanctions aimed to limit the countries resources directly, no international monetary transactions were allowed between Iran and other countries, automatically restricting the resources that could be obtained by Iran (Pahlevan Sharif et al., 2018). For example, the country was not able to obtain medication and healthcare facilities due to the sanctions, resulting in the development and spread of serious diseases which forced the Iranian healthcare employees to become more resourceful when dealing with supplies, causing a strain on staff (Pahlevan Sharif et al., 2018).

In addition to Iran as a contextually unique environment for studying labour turnover, Iran is likewise a unique context for the testing of the role of psychological ownership. By testing the role of psychological ownership in Iran, this study addresses the call of Pierce et al. (2001) to examine their proposed theory of psychological ownership across contextually different samples in order to see whether there are cross‐cultural differences in the role of psychological ownership. Since Iran faces resource shortages that affect the daily functioning of an organization, employees themselves play a crucial role to maintain smooth daily functioning. Psychological ownership could therefore have a more pronounced influence on the employment of Iranian healthcare workers.

Thus, the current study aims to examine (a) the mediating role of job satisfaction in the relationship between organisational support and nurses’ turnover intentions and (b) the moderating effect of psychological ownership on the relationship between organisational support and job satisfaction among nurses in healthcare organisations in Iran.

## 2 LITERATURE REVIEW

Labour turnover and its antecedents such as turnover intention, turnover cognitions, job search intention and withdrawal behaviour (Rubenstein et al., 2015) have been broadly, but not exhaustively, studied in the psychological, managerial and economic literature, mainly focusing on the costs to organisations and employees (Hinkin & Tracey, 2000). Employees who consider leaving their job and organisation are often mentally distanced from the work environment which results in decreased performance and less efficient and effective work compared to co‐workers who do not hold the same turnover intentions (Eriksson et al., 2021; Keaveney & Nelson, 1993; Zimmerman & Darnold, 2009). Therefore, employees with turnover intentions can be costly to organisations (Cascio, 1991) not only in terms of staff morale but also the costs associated with recruitment and training.

In the context of the healthcare sector, staff recruitment, training and turnover have received previous academic attention (Ang et al., 2013; Boon & Biron, 2016; Eriksson et al., 2021; Houshmand et al., 2012; Regts & Molleman, 2013). Such research has revealed two antecedents to be the best predictors of turnover (Rubenstein et al., 2015): organisational support (El‐Akremi et al., 2014; Eriksson et al., 2021) and job satisfaction (Al‐Ahmadi, 2014; Morrell et al., 2007).

### 2.1 Organisational support

The literature defines organisational support for nursing as “a set of core attributes of a supportive work environment that are modifiable through managerial decision‐making” (Flynn, 2007, p. 201). Organisational support consists of both tangible and intangible support, where tangible organisational support includes for example work equipment and monetary resources, and intangible organisational support includes socio‐emotional support, such as a motivating and encouraging work environment (Sing et al., 2018). There is solid theoretical evidence for the relationship between organisational support and turnover intentions (Griffeth et al., 2000). The theory of organisational support (Eisenberger et al., 1986) suggests that employees react to the way in which organisations treat them and therefore the theory is centred around the employee‐organisation relationship.

Moreover, social exchange theory (Blau, 1964) explains the relationship between employees and the organisation, based on reciprocity and the return of favours (Gouldner, 1960). From this perspective, employees who perceive their work environment supportive would experience an obligation to reciprocate by providing better service to the organisation. This employee support could take the form of stronger organisational commitment and the desire to continue employment as a result of more than simply obligation, rather a commitment to help the organisation reach its targets (Campbell et al., 2013; Rhoades & Eisenberger, 2002). Also, the way employees are treated by the organisation provides them with an idea of whether the organisation is satisfied or dissatisfied with them, indicating how much the organisation cares about their well‐being (Rhoades & Eisenberger, 2002). This notion has received empirical support with a number of empirical studies revealing a negative relationship between organisational support and employee turnover intentions (Cohen & Kirchmeyer, 2005; El‐Akremi et al., 2014; Tuzun & Kalemci, 2012). As a consequence of this discussion, the following hypothesis is developed.

H 1 There is a negative relationship between organisational support and turnover intention.

### 2.2 The mediating role of job satisfaction

A large body of research has provided support on the positive relationship between organisational support and job satisfaction (Dupre & Day, 2007; Eisenberger et al., 1997; Li et al., 2020; Pahlevan Sharif et al., 2018; Stamper & Johlke, 2003). From the lens of social exchange theory (Blau, 1964), this relationship implies that employees who perceive that their organisation provides sufficient support for them to perform efficiently and effectively will respond with higher job satisfaction. Moreover, research has shown that job satisfaction is a predictor of turnover intentions (Lambert et al., 2001; Li et al., 2020; Rubenstein et al., 2018). According to social exchange theory (Blau, 1964), employees respond with positive job attitudes (i.e. lower turnover intentions) when they are satisfied with their job. In a study involving multiple samples from a variety of backgrounds, Chen et al. (2011) found that higher levels of job satisfaction result in less likelihood to quit. Job satisfaction was also found to mediate the relationship between an organization and work characteristics with an intention to stay (Li et al., 2020). Specifically, in the healthcare sector, Regts and Molleman (2013), in their study on Dutch hospital nurses, provided support for the mediating role of job satisfaction in the relationship between perceived interpersonal citizenship behaviour and turnover intentions. This research consolidates existing literature on the negative relationship between organisational support and turnover intentions by proposing job satisfaction as a mediator in this relationship. More specifically, it is expected that organisational support is positively related to job satisfaction, which in turn negatively contributes to turnover intentions. Thus, the following hypotheses are developed.

H 2 There is a positive relationship between organisational support and job satisfaction.

H 3 There is a negative relationship between job satisfaction and turnover intention.

H 4 Job satisfaction mediates the negative relationship between organisational support and turnover intention.

### 2.3 The moderating role of psychological ownership

Psychological ownership theory implies that the relationship an employee feels toward his/her organisation can be stronger based on the degree he/she identifies him or herself with the organisation (Pierce et al., 2001). This identification is based on possession feelings of parts of the organization, such as tasks, surroundings, circumstances and colleagues. Social identity theory suggests that employees who feel that they are part of a larger whole, such as an organization, feel greater belonging, a sense of pride and self‐esteem (Tajfel & Turner, 1986). The more employees identify with an organization, based on the degree of psychological ownership, the more they feel that they have valuable possessions and exhibit positive emotions, i.e. job satisfaction (Formanek, 1991), and will try to protect these resources. The reverse is also true with the lack or loss of possessions creating a negative emotional reaction, reducing job satisfaction.

A recent study showed that psychological ownership moderated the relationship between various work‐related factors and destructive behaviour (Kong & Kim, 2017). This, along with the literature reviewed earlier on the positive relationship between organisational support and job satisfaction (Dupre & Day, 2007; Eisenberger et al., 1997; Pahlevan Sharif et al., 2018; Stamper & Johlke, 2003), suggest that the organisational support‐job satisfaction link would be stronger if employees feel greater identification to the organization through feelings of psychological ownership. In other words, the direct relationship between organisational support and job satisfaction would be stronger for employees with high levels of psychological ownership. Thus, the indirect relationship between organisational support and turnover intentions, mediated by job satisfaction, would be stronger for employees with high levels of psychological ownership. Likewise, it is expected that employees who experience lower psychological ownership for their organization may exhibit lower feelings of satisfaction which could in return affect the degree of turnover intentions. Therefore, the following hypothesis is developed.

H 5 Psychological ownership moderates the relationship between organisational support and job satisfaction, so that psychological ownership strengthens the positive relationship between organisational support and job satisfaction.

## 3 METHOD

A cross‐sectional research design was used to test the hypotheses. The data used in this study is part of a broader project on nurses’ outcomes collected using a survey questionnaire in two public hospitals in Amol, Iran from February to March 2017. Using a purposive sampling technique, 341 nurses participated in the study. The inclusion criteria were (a) working experience of more than six months and (b) no recent record of severe stress. In line with good ethical practice, the study protocol and procedures were approved by the ethical committee of Mazandaran University of Medical Sciences with the purpose of the study explained to the participants by the chief matron of the hospital. All participants were assured that the questionnaires were anonymous and participation in the study was voluntary.

### 3.1 Data analysis

Descriptive statistics were used to compute the mean and standard deviation of continuous variables, and the frequency and percentage of categorical variables. The measurement model was assessed using covariance‐based structural equation modelling and AMOS version 24. Several model fit indices were used to evaluate the model fit (Sharif et al., 2018). Items with a factor loading of below 0.5 were excluded. Cronbach's alpha >0.7 was used as an indication of good internal consistency. To establish reliability, composite reliability and maximal reliability of the constructs should be >0.7. For convergent validity, this study used composite reliability of >0.7 and average variance extracted (AVE) of 0.5 and above. To establish discriminant validity, a maximum shared variance of each construct should be less than its AVE (Pahlevan Sharif & Sharif Nia, 2018). Then, a latent variable score of the constructs was computed and the moderated mediation model was tested using SPSS version 20 and PROCESS version 2.04 (Model 7). All tests were two‐tailed and a *p*‐value of <0.05 was considered to be statistically significant.

### 3.2 Instruments

#### 3.2.1 *Organisational* *support*

To measure organisations’ support for nursing practice, following Pahlevan Sharif et al. (2018) and Flynn (2007), this study used the 9‐item Organisational Support for Nursing subscale of the Nursing Work Index–Revised (Aiken et al., 2002). Different aspects of nursing work environment features such as resource adequacy, nurse autonomy, nurse control and nurse‐physician relationships have been addressed by this sub‐scale. Various studies have confirmed the validity of the sub‐scale (Flynn, 2003, 2007; Flynn et al., 2005; Pahlevan Sharif et al., 2018). Each item was recorded on a five‐point Likert scale ranging from 1 (strongly disagree) to 5 (strongly agree). Cronbach's alpha was 0.803, indicating high reliability.

#### 3.2.2 *Job* *satisfaction*

Using four questions, participants were asked to rate their satisfaction with their current job on a seven‐point Likert scale ranging from 1 (strongly disagree) – 7 (strongly agree) (Shaver & Lacey, 2003). Cronbach's alpha of the construct was 0.809 indicating good reliability.

#### 3.2.3 *Turnover* *intention*

Four questions were asked on a seven‐point Likert scale from 1 (Strongly disagree) – 7 (strongly agree) to measure participants’ turnover intention (Tepper et al., 2009). Cronbach's alpha of 0.828 indicated good reliability.

#### 3.2.4 *Psychological* *ownership*

This study used the adapted version of job psychological ownership (Mayhew et al., 2007) which was originally developed by Van Dyne and Pierce (2004). Using five questions, the extent to which participants felt a sense of ownership for the job that they performed as a nurse was assessed on a seven‐point Likert scale from 1 (strongly disagree) – 7 (strongly agree) (Mayhew et al., 2007). Cronbach's alpha of 0.802 showed good reliability for the construct.

#### 3.2.5 Ethics approval and consent to participate

Before data collection, approval was sought from the Ethics Committee of Mazandaran University of Medical Sciences [Code: IR.MAZUMS..REC.1399.6957]. Ethical considerations (i.e., informed consent, objective and procedures of the study, confidentiality and non‐disclosure of personal information, prevention of financial burden on the participants, the right to withdraw from participation at any point in the study) were explained to the participants.

## 4 RESULTS

The results of conducting confirmatory factor analysis (CFA) are reported in Table 1. The last reversed question of psychological ownership and three items of organisational support were removed due to their weak factor loading. The revised measurement model showed a good fit [*χ* ^2^(98) = 197.909, *p* <.001, *χ* ^2^/*df* = 2.019, goodness of fit index (GFI) = 0.932, comparative fit index (CFI) = 0.960, incremental fit index (IFI) = 0.960, Tucker–Lewis Index (TLI) = 0.951, relative fit index = 0.907, normed fit index (NFI) = 0.924, standardized root mean square residual (SRMR) = 0.052 and root mean square error of approximation (RMSEA) (90% confidence interval (CI)) = 0.055 (0.044–0.066)]. All factor loadings were >0.5 (ranged from 0.547–0.948) and statistically significant (*z*‐value ranged from 8.834–26.403). All constructs showed good reliability (composite reliability ranged from 0.804–0.895 and maximal reliability ranged from 0.918–0.973). The AVE of the constructs ranged from 0.409–0.688. Among them, the AVE of organisational support was <0.5 which could be due to the conservativeness of AVE measure. Thus, this study used composite reliability >0.7 as an indication of good convergent validity (Pahlevan Sharif et al., 2018; Pahlevansharif & Sharif Nia, 2018). The maximum shared variance (ranging from 0.061–0.391) of each construct was less than its respective AVE that established discriminant validity of all constructs.

**TABLE 1:** The results of measurement model assessment

Constructs	Items	Factor loadings	Cronbach's alpha	Composite reliability	Maximal reliability	Average variance extracted	Maximum shared variance
Organizational support	OS 3	0.562***	0.803	0.804	0.940	0.409	0.391
	OS 4	0.554***					
	OS 5	0.620***					
	OS 6	0.678***					
	OS 8	0.753***					
	OS 9	0.649***					
Psychological ownership	PO 1	0.884***	0.802	0.895	0.968	0.688	0.061
	PO 2	0.907***					
	PO 3	0.922***					
	PO 4	0.547***					
Intention to leave	IL 5	0.653***	0.828	0.840	0.918	0.642	0.061
	IL 6	0.948***					
	IL 7	0.776***					
Job Satisfaction	JS 1	0.665***	0.809	0.824	0.973	0.613	0.391
	JS 3	0.908***					
	JS 4	0.756***					

*** *p* <.001

Table 2 shows the results of performing Pearson correlation analysis on all constructs that have been replaced with their latent variable score. In line with the hypotheses, both organisational support and psychological ownership were positively related to job satisfaction and negatively linked to turnover intention. Also, job satisfaction was negatively related to turnover intention.

**TABLE 2:** The results of Pearson correlation analysis

	Mean	*SD*	[2]	[3]	[4]
[1] Organizational support	2.150	0.654	0.052	0.708**	−0.120*
[2] Psychological ownership	3.742	0.936		0.136*	−0.263**
[3] Job satisfaction	2.654	1.121			−0.236**
[4] Intention to leave	1.680	1.015			

* *p* <.05,

** *p* <.01.

**FIGURE 1:**
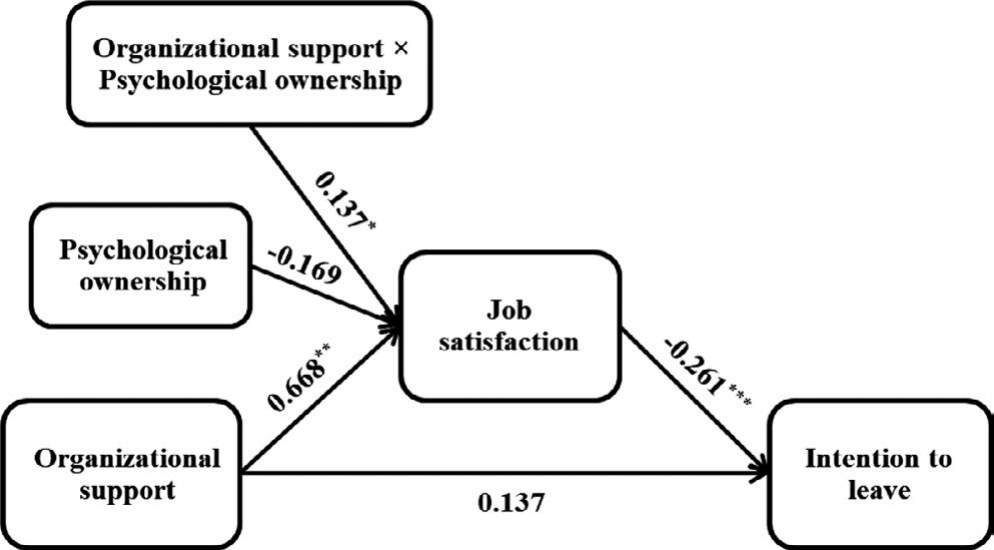
The model with the results. ^*^ *p* < .05, ^**^ *p* < .01, ^***^ *p* < .001, The results were controlled for the effects of gender, age, and education level

Table 3 and Figure 1 show the results of the structural model assessment after controlling for the effects of gender, age and education level. There was a significant negative relationship between organisational support and turnover intention (*b* = −0.174, *p* <.05) supporting H1. The results of assessing the moderated mediation model indicated a significant positive relationship between organisational support and job satisfaction (*b* = 0.668, *p* <.01) and a significant negative relationship between job satisfaction and turnover intention (*b* = −0.261, *p* <.001), providing support for H2 and H3, respectively. Also, job satisfaction mediated the negative relationship between organisational support and turnover intention (*b* = −0.308, *p* <.01) which supported H4.

**TABLE 3:** The results of the structural model assessment

Paths	Unstandardized coefficient	95% Confidence intervals
Total effect model		
Organizational support →Intention to leave	−0.174*	[−0.348, −0.001]
Moderated mediation model		
Organizational support →Job satisfaction	0.668**	[0.172, 1.163]
Psychological ownership →Job satisfaction	−0.169	[−0.459, 0.121]
Organizational support × Psychological ownership →Job satisfaction	0.137*	[0.010, 0.265]
Job satisfaction →Intention to leave	−0.261***	[−0.398, −0.125]
Organizational support →Intention to leave	0.137	[−0.098, 0.373]
Organizational support →Job satisfaction →Intention to leave | Psychological ownership	−0.308**	[−0.510, −0.126]
Organizational support →Job satisfaction →Intention to leave | Psychological ownership = 2.790	−0.275**	[−0.464, −0.106]
Organizational support →Job satisfaction →Intention to leave | Psychological ownership = 4.666	−0.342***	[−0.555, −0.142]

Note

The results were controlled for the effects of gender, age and education level.

* *p* <.05,

** *p* <.01,

*** *p* <.001.

**FIGURE 2:**
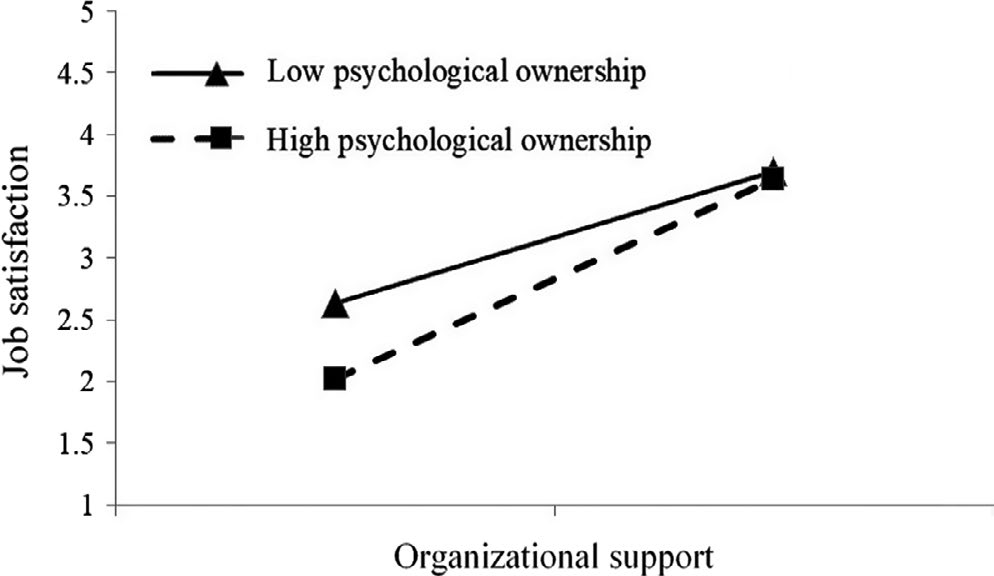
The moderating effect of psychological ownership on the relationship between organizational support and job satisfaction

Moreover, the significant positive relationship between the interaction of psychological ownership and organisational support with job satisfaction (*b* = 0.668, *p* <.01) revealed that psychological ownership strengthens the positive relationship between organisational support and job satisfaction supporting H5. Figure 2 shows the relationship between organisational support and job satisfaction for low and high levels of psychological ownership. The results also showed that the aforementioned mediation relationship when psychological ownership was one standard deviation below the mean (*b* = −0.275, *p* <.01) and one standard deviation above the mean (*b* = −0.342, *p* <.001) was positive and statistically significant. The model explained 51.70% of the variance of job satisfaction and 9.85% of the variance of turnover intention.

## 5 DISCUSSION

The results regarding the first hypothesis showed a negative relationship between organisational support and turnover intention (H1). Consistent with the past literature, the results indicated that the key role of an organisation is the establishment and maintenance of a supportive environment where employees are furnished with both tangible and intangible supports (Flynn, 2007; Rhoades & Eisenberger, 2002; Sing et al., 2018). These findings also support previous research that showed employees’ fidelity to the organisation is contingent on the services and support they receive from the organisation (Gouldner, 1960). In other words, the more support the employees felt, the less likely they were to leave the organisation (Campbell et al., 2013; Cohen & Kirchmeyer, 2005; El‐Akremi et al., 2014; Rhoades & Eisenberger, 2002). Nurses’ favourable feeling towards the organisation, most likely is formed by a combination of inter‐related factors including adequate staffing, the support of a nurse by the manager(s) and a good nurse–physician relationship (Pahlevan Sharif et al., 2018). This favourable emotion and positive relationship with the organisation combined with the support mechanisms in place, engender more job satisfaction and increase the likelihood that employees would stay with their current employer.

The research also confirmed a positive relationship between organisational support and job satisfaction (H2). This result is consistent with the literature which argues that employees’ satisfaction with the organisation is a function of the support they receive from the organisation (Dupre & Day, 2007; Sharif Nia et al., 2021; Stamper & Johlke, 2003). Nurses perceiving such organisational support, less likely would leave their job (Chen et al., 2011; Lambert et al., 2001; Rubenstein et al., 2018), which supports the third hypothesis proposing a negative correlation between job satisfaction and turnover intention (H3). Taken together, these findings suggest the mediating role of job satisfaction in the organisational support‐turnover intention link (H4). These results lend support to the studies that identified job satisfaction as a mediator in the mechanisms explaining turnover intention (Li et al., 2020; Regts & Molleman, 2013). The results suggest that organisations should prioritise employees’ satisfaction to ultimately improve organization performance.

Moreover, this study supported H5 which proposed that psychological ownership strengthens the positive relationship between organisational support and job satisfaction. In other words, when employees are psychologically devoted to the organisation, they are more likely to be engaged and satisfied with their job (Formanek, 1991; Pierce et al., 2003; Schirle et al., 2019). This is because the feeling of possessiveness and being mentally tied to the organisation will create and reinforce a feeling of being committed to the organisation. This implies the need to cultivate a sense of being psychologically attached to the organisation as it can help employees realise their self‐worth and contribution to the workplace, addressing their socio‐emotional needs and their connectedness to their job.

Organisations may not afford all expected tangible supports and services in a short timeline. However, to ensure their employees remain with the organisation, they could capitalise on their employees’ cognitive and affective feelings of being mentally attached to the organisation which can be attained within a relatively short period of time. Another contribution of this study is supporting the moderating role of psychological ownership on the mediating effect of job satisfaction in the organisational support‐turnover intention link that establishes the inclusive mechanism explaining how organisational support leads to employees’ turnover intention. For employees with higher levels of psychological ownership, the negative impact of organisational support on turnover intention, mediated by job satisfaction, is more well‐established, suggesting that the turnover intention is driven by employees’ level of psychological ownership.

The current study is not without limitations. Firstly, the research only surveyed nursing professionals at two public hospitals located in an urban area of Iran. This was not considered a major limitation given the research wished to study nurses in particular and was the initial part of a larger study involving a number of healthcare institutions across different parts of Iran. Secondly, the cross‐sectionality of the study limits the conclusion on the causality of the relationships. Thus, future longitudinal studies are recommended. The third limitation was using a unidimensional scale to measure psychological ownership. To capture more comprehensive results, future studies can employ multi‐dimensional scales of psychological ownership consisting of self‐efficacy, self‐identity and belongingness.

## 6 CONCLUSION

The aims of this study were two‐fold, to examine the relationship between organisational support and nurses’ turnover intentions with the mediating role of job satisfaction and to investigate the moderating role of psychological ownership in the relationship between organisational support and job satisfaction. The research consolidated existing literature in the area of turnover intentions (Griffeth et al., 2000; Pahlevan Sharif et al., 2018; Rubenstein et al., 2018). The study also took research forward in terms of addressing the gap in the literature regarding turnover research across different cultures (Rubenstein et al., 2018).

The research found that organisational support, job satisfaction and psychological ownership impact healthcare professionals’ turnover intention. One would expect a correlation between organisational support and job satisfaction and as a consequence, an intention to remain with the healthcare employer. What was interesting was the moderating role of psychological ownership in the relationship between organisational support and job satisfaction, so that psychological ownership strengthened the positive relationship between organisational support and job satisfaction. With regards to further research, it is the intention of the researchers to undertake a wider study across other Middle Eastern hospitals to investigate commonalities and themes to allow the findings to be more readily generalised. In terms of other future research, it is proposed to investigate and contrast the perspectives from other healthcare professionals: support staff; doctors and management, contrasting perspectives of those who are currently still in the healthcare profession with those who have left the employ of the hospital. Gaining the perspectives of all these stakeholders would provide a more holistic view of turnover intentions and the interactivity between psychological ownership, organisational support, job satisfaction and turnover intentions.

## CONFLICT OF INTEREST

I hereby acknowledge that authors have no conflict of interest.

## AUTHOR CONTRIBUTION

All authors made a substantial contribution to writing of the paper draft and met the four criteria for authorship recommended by the International Committee of Medical Journal Editors.

## Data Availability

The datasets used and/or analyzed during the current study available from the corresponding author on reasonable request.
